# Hormone Impact: BPA Linked to Altered Gene Expression in Humans

**DOI:** 10.1289/ehp.119-a524b

**Published:** 2011-12-01

**Authors:** Julia R. Barrett

**Affiliations:** Julia R. Barrett, MS, ELS, a Madison, WI–based science writer and editor, has written for *EHP* since 1996. She is a member of the National Association of Science Writers and the Board of Editors in the Life Sciences.

Urinary metabolites of bisphenol A (BPA), a widely used component of polycarbonate plastics and epoxy resins, serve as bio-markers for exposure to the chemical and are detectable in more than 90% of indi-viduals tested in the United States and Europe. Studies to date suggest positive associations between BPA and cardio-vascular disease, diabetes, and reproductive and developmental abnormalities in humans, although further research is needed to confirm these findings. Recent studies have shown links between BPA and changes in total testosterone concentrations and altered estradiol:testosterone ratio in men, but evidence for a mechanism behind such links has been lacking. A new study now links BPA exposure with altered expression of estrogen- and androgen-responsive genes in humans **[*EHP* 119(12):1788–1793; Melzer et al.]**.

The InCHIANTI study—a prospective study of mid- and late-life morbidity risk factors among 1,453 participants in Chianti, Italy—provided the data for the current study. A subset of 96 men provided same-day blood and urine samples in 2008–2009. Urine samples were analyzed for concentrations of BPA, and blood leukocytes were used for transcript analysis of six estrogen- and androgen-responsive genes: *ESR1*, *ESR2*, *ESRRA*, *ESRRB*, *ESRRG*, and *AR*. These genes code nuclear hormone receptors involved in the control of developmental and physiological pathways shown to be activated by BPA in laboratory studies.

Urinary BPA concentrations ranged from 0.73 to 56.94 ng/mL and were positively associated with increased expression of *ESR2* and *ESRRA* based on models adjusted for potential confounding factors. Transcripts for other genes were either not detected (*ESRRG* ) or were not associated with BPA concentrations (*ESR1*, *ESRRB*, and *AR*). Mean expression of *ESR2* and *ESRRA* increased by 65% and 38%, respectively, in the highest versus lowest BPA exposure tertile.

The implications of altered gene expression in blood leukocytes are unknown, and this measure has not been validated as a surro-gate measure of effects on hormone-responsive gene expression. However, the results suggest that BPA is bioactive in humans, and the authors argue that the potential link between exposure, hormone signaling, and related disorders is biologically plausible. For example, estrogen receptor β, coded by *ESR2*, plays a significant role in maintaining the structure and function of tissues in the cardiovascular and central nervous systems.

The cross-sectional design, lack of distinction between free and conjugated BPA in urine samples, and possible unidentified confounding factors are limitations of the study. Additional research is needed to confirm the findings and further investigate gene expression changes and effects of BPA exposure in other estrogen-regulated target tissues.

